# Carbon dioxide and trace oxygen concentrations impact growth and product formation of the gut bacterium *Phocaeicola vulgatus*

**DOI:** 10.1186/s12866-023-03127-x

**Published:** 2023-12-07

**Authors:** Laura Keitel, Kristina Braun, Maurice Finger, Udo Kosfeld, Stanislav Yordanov, Jochen Büchs

**Affiliations:** Chair of Biochemical Engineering (AVT.BioVT), RWTH Aachen University, Forckenbeckstraße 51, 52074 Aachen, Germany

**Keywords:** *Phocaeicola (Bacteroides) vulgatus*, Gut bacteria, Anaerobic fermentation, Short chain fatty acids (SCFA), Carbon dioxide optimum, Oxygen tolerance

## Abstract

**Background:**

The promising yet barely investigated anaerobic species *Phocaeicola vulgatus* (formerly *Bacteroides vulgatus*) plays a vital role for human gut health and effectively produces organic acids. Among them is succinate, a building block for high-value-added chemicals. Cultivating anaerobic bacteria is challenging, and a detailed understanding of *P. vulgatus* growth and metabolism is required to improve succinate production. One significant aspect is the influence of different gas concentrations. CO_2_ is required for the growth of *P. vulgatus.* However, it is a greenhouse gas that should not be wasted. Another highly interesting aspect is the sensitivity of *P. vulgatus* towards O_2_. In this work, the effects of varying concentrations of both gases were studied in the in-house developed Respiratory Activity MOnitoring System (RAMOS), which provides online monitoring of CO_2_, O_2,_ and pressure under gassed conditions. The RAMOS was combined with a gas mixing system to test CO_2_ and O_2_ concentrations in a range of 0.25-15.0 vol% and 0.0-2.5 vol%, respectively.

**Results:**

Changing the CO_2_ concentration in the gas supply revealed a CO_2_ optimum of 3.0 vol% for total organic acid production and 15.0 vol% for succinate production. It was demonstrated that the organic acid composition changed depending on the CO_2_ concentration. Furthermore, unrestricted growth of *P. vulgatus* up to an O_2_ concentration of 0.7 vol% in the gas supply was proven. The viability decreased rapidly at concentrations larger than or equal to 1.3 vol% O_2_.

**Conclusions:**

The study showed that *P. vulgatus* requires little CO_2_, has a distinct O_2_ tolerance and is therefore well suited for industrial applications.

**Supplementary Information:**

The online version contains supplementary material available at 10.1186/s12866-023-03127-x.

## Background

The largest population of bacteria in the human body inhabits the intestine, with about 10^11–12^ organisms per mL of colonic contents [1, 2]. The bacterial microbiota of the intestine facilitates the maturation of the immune system, the development of the gut and protects against colonization by pathogens. It also supports the human metabolism by breaking down indigestible polysaccharides into nutrients, vitamins, co-factors, amino acids, and short-chain fatty acids (SCFAs) [3–5]. The most common phylum in the human gut is *Bacteroidota* [2, 6]. Species of *Bacteroidota* achieve high yields of organic acids [7–9] and can be genetically modified [10, 11]. *Phocaeicola vulgatus*, initially classified as *Bacteroides vulgatus* [12], is one of the most abundant bacteria within the phylum of *Bacteroidota* [6]. Even though *P. vulgatus* has great potential as an industrial platform organism, it has not yet been used for biotechnological processes [10], because the species.

has not been sufficiently characterized in axenic culture. The cultivation of gut microbes is complex, as they are highly adapted to the gastrointestinal ecosystem [2, 13].

The gastrointestinal ecosystem is an environment that offers CO_2_ in abundance, as the gas is a by-product of anaerobic fermentation. The CO_2_ is then taken up by enterocytes or utilized by other microorganisms [14]. Another aspect of the intestine is the changing O_2_ level. The gut epithelium is supplied with O_2_ by the vasculature, and the bulk of the lumen is essentially anoxic [15]. Due to the O_2_ gradient, even strict anaerobic gut bacteria need response mechanisms, if they encounter higher O_2_ concentrations in the intestine or escape the gut environment. Species of *Bacteroidota* are classified as opportunistic pathogens with a broad range of oxygen tolerance, capable of infecting oxygenated tissues [2]. O_2_ can diffuse into the bacterial cells and inactivate enzymes with a radical in the active center [15]. Another common mechanism of O_2_-induced damage includes the formation of reactive oxygen species (ROS) in the form of superoxide and hydrogen peroxide [16]. ROS are formed, when molecular O_2_ oxidizes reduced metals and thiols. Anaerobes protect themselves from ROS with the same defensive tactics initially identified in aerobes [15]. The arsenal of the genus *Bacteroides* against O_2_-induced damage contains e.g., peroxidases, rubrerythrins, and catalases [15]. In an oxygenated environment, *Bacteroidota* switch to a stationary-like state to protect themselves from damage by ROS. In this condition, the translation of biosynthesis genes is downregulated, and growth is impaired [17, 18]. Furthermore, on a transcriptional level, downregulation of potential ROS-producing enzymes, such as fumarate reductase, occurs [19]. Fumarate reductase is responsible for the reduction of fumarate to succinate in *P. vulgatus* [3]. The genetically related *Bacteroides fragilis* species can also express cytochrome *bd* oxidase [20]. In this way, O_2_ serves as a terminal electron acceptor in the respiratory chain. Ultimately, cytochrome *bd* oxidase can stimulate O_2_-dependent growth in micro-aerobic conditions.

The carbon metabolism is a significant aspect of a better understanding of *P. vulgatus*. One of the three glycolytic pathways is used in related *Bacteroides* to obtain phosphoenolpyruvate (PEP), a key metabolite in glycolysis. PEP is then converted to products such as organic acids and gases [3]. This conversion is done using anaerobic respiration and fermentation via oxaloacetate, malate, and fumarate [3]. *Bacteroides* use anaerobic respiration, since it is generally more efficient than fermentation [3]. The main products of the anaerobic respiration of *Bacteroides* are acetate, propionate, succinate, lactate, formate, CO_2_, and H_2_ [3]. The high CO_2_ levels in the gut are advantageous for anaerobic respiration [3]. Thereby, *Bacteroides* can establish a primitive electron transport chain based on reducing fumarate to succinate [3]. As a result, CO_2_ is fixed to fumarate, and the bacterium can regenerate CO_2_ from succinate under CO_2_-limiting conditions [3]. Through this process, propionate can be produced [3]. Additionally, lactate is formed by reducing pyruvate via lactate dehydrogenase [10]. *Prevotella copri*, another *P. vulgatus*-related species, can convert pyruvate to formate, CO_2_, Fd_red_ (possible site for hydrogen formation), and acetyl-CoA, which is converted in the next step to acetate [21].

The SCFAs acetate, propionate, succinate, formate, and lactate, a short-chain hydroxy fatty acid, denoted as an SCFA in this study, are the main products of *P. vulgatus*. SCFAs are important for gut microbes, to regulate the production of redox equivalents in the anaerobic environment of the intestine [22]. Moreover, SCFAs benefit the human host and serve as signal molecules or energy substrates [23, 24]. Currently, most SCFAs for the chemical industry are produced based on fossil fuels. However, as *Bacteroidota* produce numerous SCFAs, there is the potential for a sustainable production.

Although only limited efforts for characterization of *P. vulgatus* in terms of CO_2_ requirement and O_2_ tolerance have been conducted so far, Franke and Deppenmeier [21] and Reilly [25] have shown that *P. vulgatus* requires CO_2_ or bicarbonate supplementation for growth. However, as the study of Franke and Deppenmeier [21] focused on *P. copri*, they found a more pronounced CO_2_ dependency of *P. copri*, compared to *P. vulgatus*. Furthermore, Baughn and Malamy [20] stated that *P. vulgatus* could cope with oxygen concentrations of 0.03 vol% without suffering damage. However, the optimum CO_2_ level and maximal oxygen tolerance still need to be unraveled.

This study aims to advance the characterization of *P. vulgatus* under anaerobic cultivation conditions, determining the CO_2_ requirement and O_2_ tolerance for growth and organic acid production. The characterization was conducted utilizing the Respiration Activity MOnitoring System (RAMOS). The RAMOS is a small-scale shaken cultivation system that, in contrast to traditional serum flasks, allows for non-invasive online measurement of CO_2_, O_2,_ and pressure [26–28]. Furthermore, the influence of different gas concentrations was determined by combining the RAMOS with a gas mixing system. Thereby, up to four different gas streams were supplied to the RAMOS. This study determines the feasibility of *P. vulgatus* as an efficient organic acid producer under different influences of CO_2_ and O_2_.

## Results

### Influence of CO_2_ on growth and acid production

**Fig. 1:**
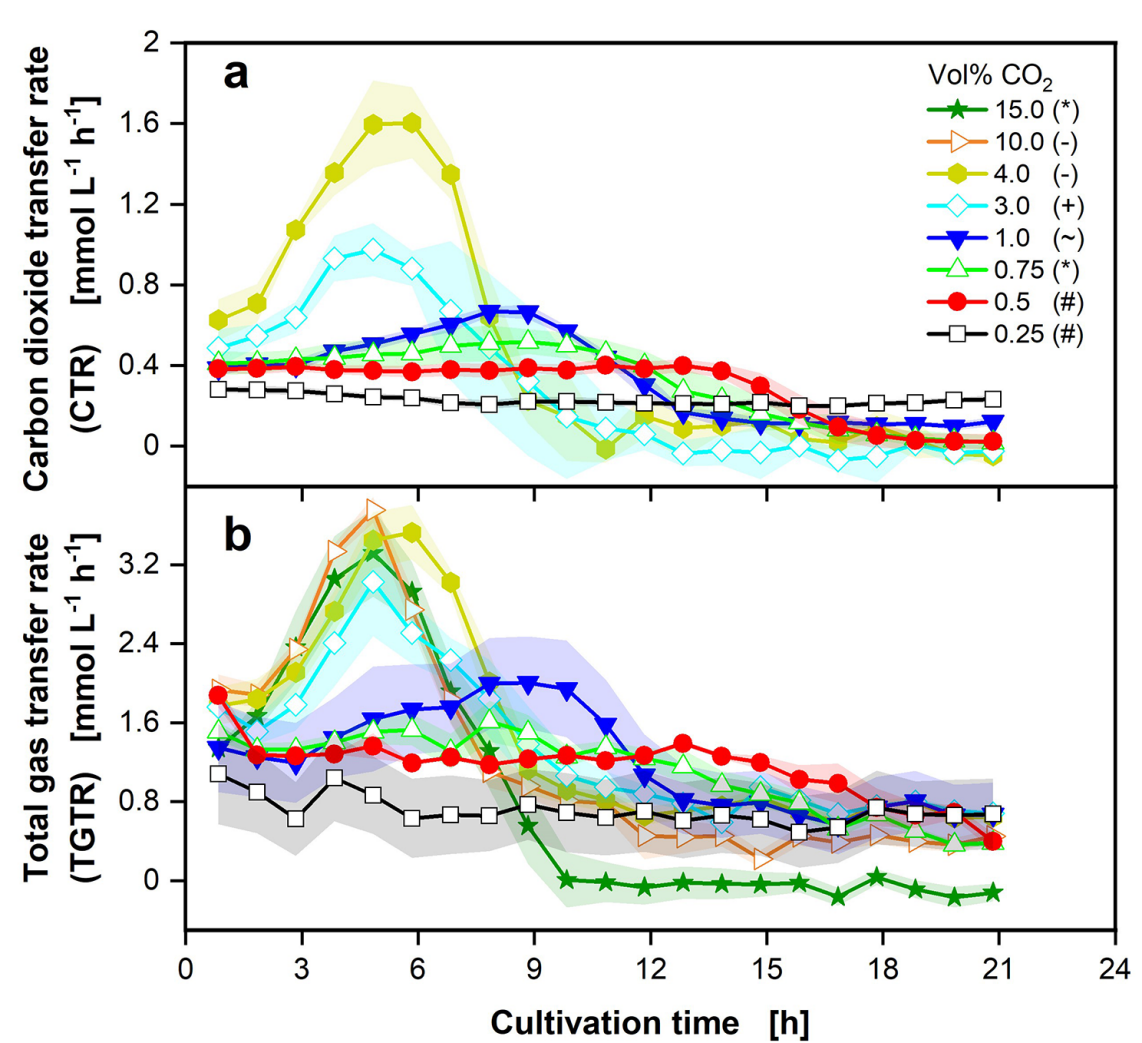
Effect of different CO_2_ concentrations on gas transfer rates of *P. vulgatus* shake flask cultivations. Online data of (**a**) carbon dioxide transfer rate (CTR) and (**b**) total gas transfer rate (TGTR). Shadows indicate standard deviations of four biological replicates. Measurement of CO_2_ was not possible above 5.0 vol% CO_2_ in the ingas, due to limited sensor range. Different successively conducted experimental runs are indicated by different symbols in the legend (*,-,+,~,#). Experimental setup is illustrated in Fig. 5a. Hydrogen transfer rate (HTR) plotted over CTR corresponding to this experiment can be found in Fig. S1. Medium: DMM-G, c_Glucose_ = 6 g L^− 1^, c_buffer_ = 50 mM MOPS, T = 37 °C, n = 100 rpm, V_L_ = 50 mL, initial OD_600nm_ = 0.2, initial pH after inoculation = 6.9–7.15, vvm = 0.2 min^− 1^, different gas mixtures of CO_2_ in N_2_, as indicated in legend

In the first set of experiments, the influence of different concentrations of CO_2_ in the gas supply on the cultivation of *P. vulgatus* in shake flasks was tested. During the experiments, the CO_2_ concentration in the gas supply varied between 0.25 and 15.0 vol% with the aid of the gas mixing system. For these cultivations, online gas transfer rates are depicted in Fig. 1.

No carbon dioxide transfer rate (CTR) is displayed for the CO_2_ concentrations of 10.0 and 15.0 vol%, as the limit of the CO_2_ sensors used in this study was reached at 5.0 vol%. The CTR curves in Fig. 1a indicate different values at the first measurement point after 1 h, depending on the CO_2_ concentration in the gas supply. For 0.25 vol% CO_2_ (black squares) in the gas supply, no substantial increase or decrease in the CTR is visible throughout the cultivation. The following concentration of 0.5 vol% CO_2_ (red circles) slightly increases, before decreasing to 0 mmol L^− 1^ h^− 1^. At 0.75 (green triangles) and 1.0 vol% (blue inverted triangles) CO_2_ in the gas supply, the CTR maximum rises with increasing CO_2_ concentration and is attained after 8.8 h. For 3.0 vol% CO_2_ (light blue diamonds), the CTR maximum is higher than the maximum of 1.0 vol%, but CO_2_ production also increases earlier than for the lower CO_2_ concentrations, and the maximum is reached earlier. With a CO_2_ concentration in the gas supply of 4.0 vol%, the CTR maximum attains a value of 1.6 mmol L^− 1^ h^− 1^, the highest CTR maximum of all conditions. In conclusion, the higher the CO_2_ concentration in the gas supply up to the highest measured concentration of 4.0 vol%, the higher the CTR maximum. Figure 1b presents the different total gas transfer rate (TGTR) progressions. Generally, the same trends represented for the CTR maxima can be seen for the TGTR maxima. The higher the CO_2_ concentration in the gas supply, the higher the TGTR maxima. This trend continues until a CO_2_ concentration of 10.0 vol% (orange triangles) is obtained. However, for 15.0 vol% CO_2_ (green stars), the TGTR maximum is slightly lower, but with a higher standard deviation. In general, the CTR peaks are lower than the TGTR peaks. The highest measurable CTR maximum reaches 1.6 mmol L^− 1^ h^− 1^, while the corresponding TGTR maximum attains 3.5 mmol L^− 1^ h^− 1^. CO_2_ only contributes between 27.2 and 45.7% to the total gas production, depending on the CO_2_ concentration in the gas supply.

**Fig. 2:**
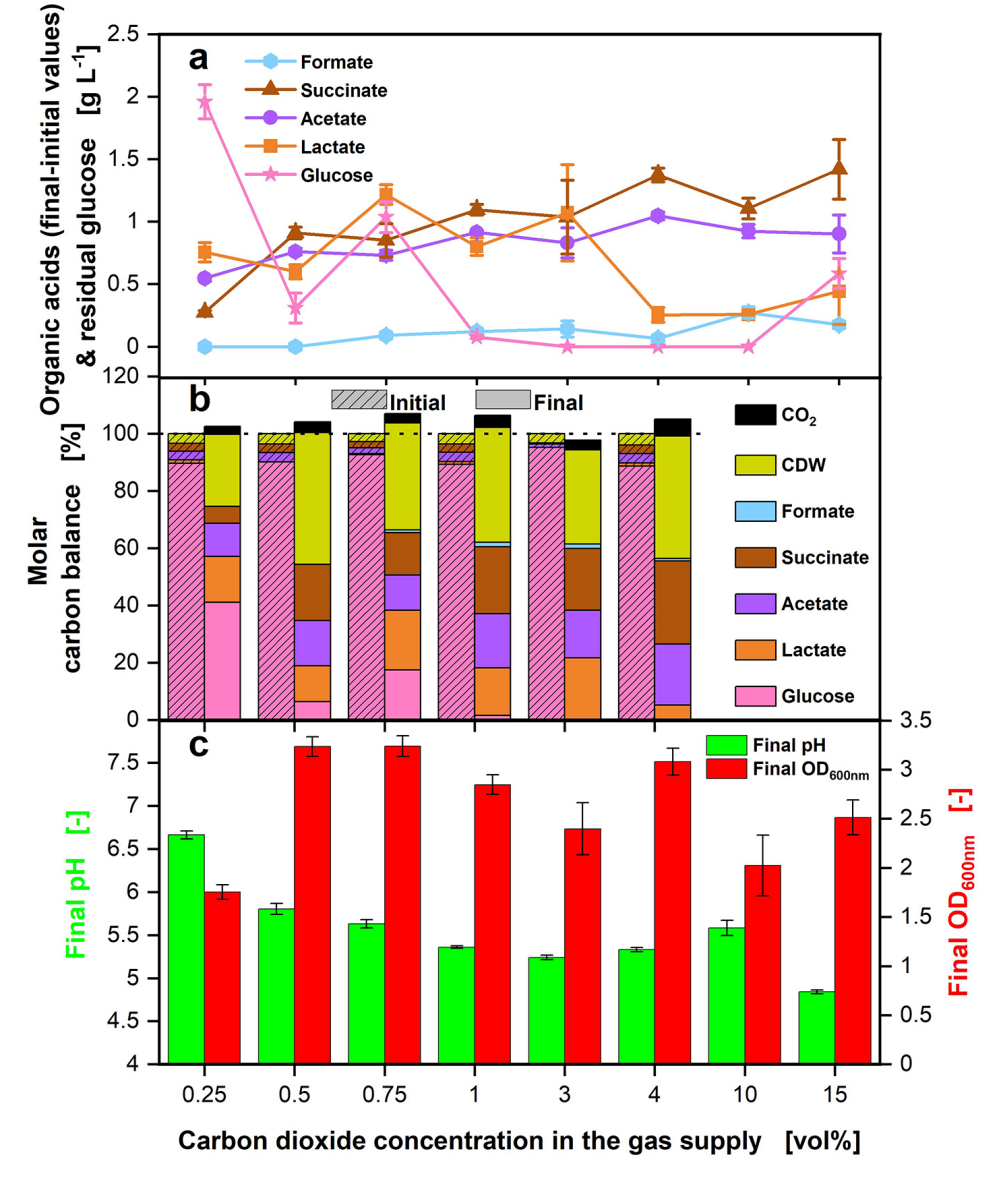
Effect of different CO_2_ concentrations on offline data of *P. vulgatus* shake flask cultivations. These data refer to the experiment shown in Fig. 1. Offline data of (**a**) HPLC analysis of produced organic acids, including formate, succinate, acetate and lactate and remaining glucose from four biological replicates with standard deviation. (**b**) Carbon balance in % as function of the CO_2_ concentration in the gas supply. The start of the fermentation was set to 100%. No carbon balance is calculated for 10.0 and 15.0 vol% CO_2_, as CO_2_ could not be measured in this range. Initial samples were drawn after inoculation. (**c**) Final OD_600nm_ and final pH from four biological replicates with standard deviation. Experimental setup is illustrated in Fig. 5a. Medium: DMM-G, c_Glucose_ = 6 g L^− 1^, c_buffer_ = 50 mM MOPS, T = 37 °C, n = 100 rpm, V_L_ = 50 mL, initial OD_600nm_ = 0.2, initial pH after inoculation = 6.9–7.15, vvm = 0.2 min^− 1^, different gas mixtures of CO_2_ in N_2_, as indicated in legend

In Fig. 2, the offline data of the cultivation is shown. High-performance liquid chromatography (HPLC) measurements were performed for the key metabolites formate, succinate, acetate, lactate, and glucose. Propionate could not be detected in these experiments. Formate (light blue hexagons), succinate (brown triangles), and acetate (purple circles) production rise with increasing CO_2_ concentration in the gas supply until 4.0 vol% (Fig. 2a). Lactate production (orange squares) is approximately two-fold higher with lower CO_2_ concentrations of up to 3.0 vol% in the gas supply and decreases with higher CO_2_ concentrations. The main acids produced are succinate, acetate, and lactate, with only small amounts of formate generated. The highest total amount of SCFAs is produced at 3.0 vol% CO_2,_ whereas the lowest is formed at 0.25 vol% CO_2_. Glucose (pink stars) is completely consumed for conditions with 3.0 and 10.0 vol% CO_2_ in the gas supply. Figure 2b depicts the molar carbon balance. The molar carbon balance (calculated according to Eqs. 1–2) is closed with a maximum deviation of 8.5%. The biomass accounts for 25 to 46% of the total carbon, depending on the CO_2_ concentration in the gas supply. Moreover, low amounts of CO_2_ are formed, reaching a maximum of 5.9% of the total carbon. In Fig. 2c, the final pH values, as well as the final optical density (OD_600nm_), are displayed. The final pH values decline for increasing CO_2_ concentrations between 0.25 and 3.0 vol%. From there, the final pH values rise until 10.0 vol% and decrease again for 15 vol% CO_2_. Overall, the final pH values are low, between 4.8 and 6.7. The final OD_600nm_ reaches the lowest value for 0.25 vol% CO_2_ with 1.8 and increases from there. It changes with no clear trend between 2.0 and 3.2 for the higher CO_2_ concentrations in the gas supply.

### Influence of O_2_ on growth and organic acid production

To determine the O_2_ tolerance of *P. vulgatus* in shake flasks, O_2_ concentration in the gas supply was varied between 0 and 2.5 vol%. The CO_2_ concentration in the gas supply was kept constant at 4.0 vol%.

**Fig. 3:**
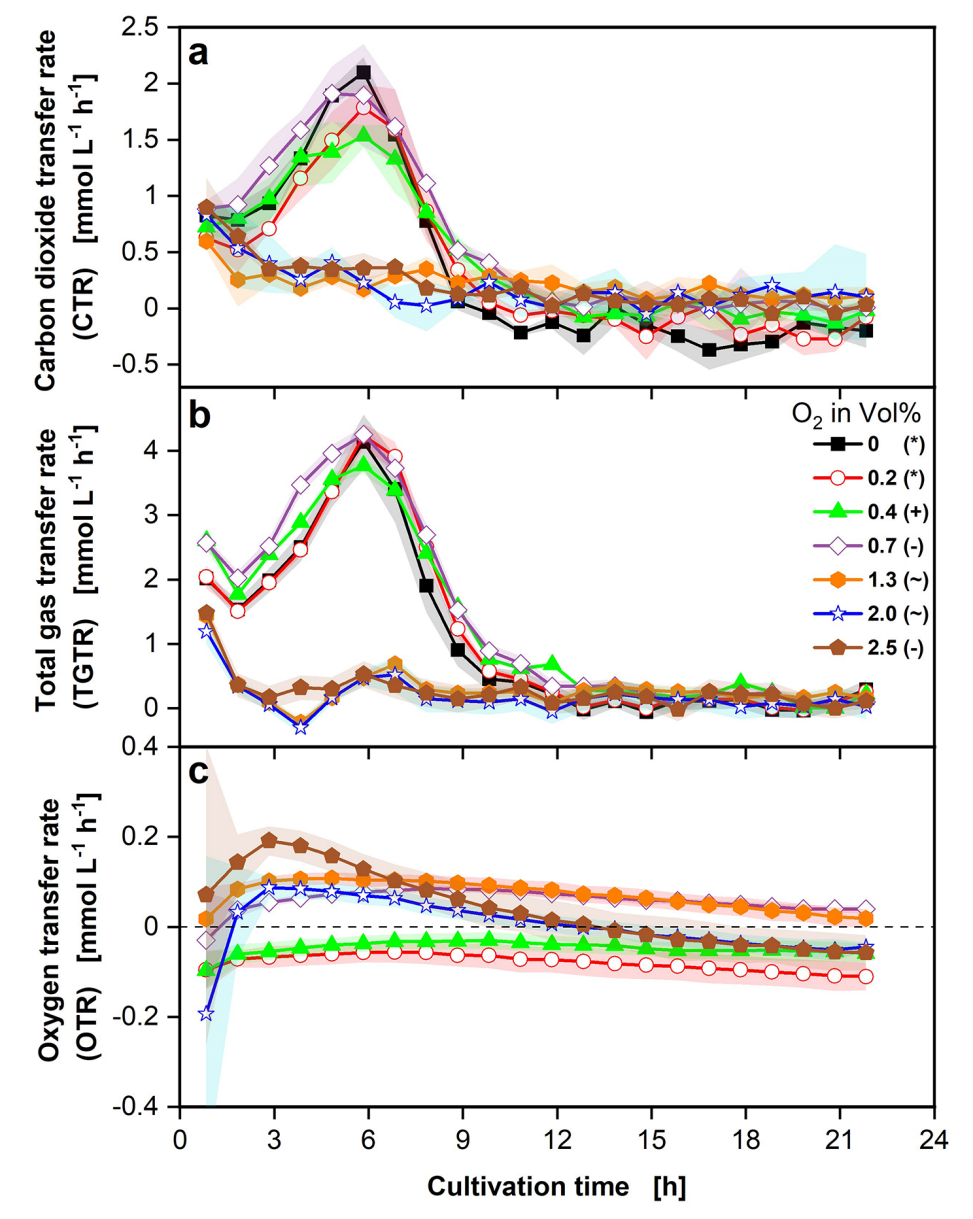
Effect of different O_2_ concentrations on gas transfer rates of *P. vulgatus* shake flask cultivations. Online data of (**a**) carbon dioxide transfer rate (CTR) and (**b**) total gas transfer rate (TGTR) and (**c**) oxygen transfer rate (OTR). Shadows indicate standard deviations of four biological replicates. Different successively conducted experimental runs are indicated by different symbols in the legend (*,+,-,~). Dashed horizontal line in (**c**) indicates an OTR of 0 mmol L^− 1^ h^− 1^. Experimental setup is illustrated in Fig. 5b. Hydrogen transfer rate (HTR) plotted over CTR corresponding to this experiment can be found in Fig. S2. Medium: DMM-G, c_Glucose_ = 6 g L^− 1^, c_buffer_ = 50 mM MOPS, T = 37 °C, n = 100 rpm, V_L_ = 50 mL, initial OD_600nm_ = 0.29, initial pH after inoculation = 6.9–7.1, vvm = 0.2 min^− 1^, different gas mixtures of O_2_ & 4% CO_2_ in N_2_, as indicated in the legend

In Fig. 3, the online gas transfer rates are shown. The CTR curves in Fig. 3a start at approximately the same value after 1 h for all tested O_2_ concentrations. The CTR curves of the lower O_2_ concentrations between 0 vol% (black squares) and 0.7 vol% (purple diamonds) rise until reaching their maximum after 6 h, with no distinct trend between the four concentrations. The CTR curves of higher O_2_ concentrations between 1.3 (orange hexagons) and 2.5 vol% (brown pentagons) directly decline, until all curves reach 0 mmol L^− 1^ h^− 1^. The TGTR curves, depicted in Fig. 3b, display the same trends. As already observed in the previous experiments, the maxima of the TGTR curves are substantially higher than those of the CTR curves, with the TGTR attaining a maximal value of 4.3 mmol L^− 1^ h^− 1^ (0.7 vol%, purple diamonds). The corresponding CTR maxima of 0.7 vol% O_2_ reaches 1.9 mmol L^− 1^ h^− 1^. In Fig. 3c, the progression of the oxygen transfer rate (OTR) is presented for the O_2_ concentrations between 0.2 and 2.5 vol%. For 0 vol%, no calculation of the OTR was possible. The O_2_ concentrations of 0.2 (red circles) and 0.4 vol% (green triangles) remain at an OTR of approximately − 0.1 mmol L^− 1^ h^− 1^ throughout the cultivation. The higher O_2_ concentrations of 0.7 to 2.5 vol% indicate a rising OTR curve in the positive range over the first hours. 2.0 vol% (blue stars) and 2.5 vol% (brown pentagons) O_2_ reach an OTR maximum after 2.8 h, while 0.7 vol% (purple diamonds) and 1.3 vol% (orange hexagons) O_2_ conditions do not indicate a clear OTR maximum. In the following cultivation time, the OTR curves of 0.7 to 2.5 vol% O_2_ decrease until reaching about 0 mmol L^− 1^ h^− 1^ at the end of the cultivation. However, it must be noted that the detection limit of the OTR is reached here.

**Fig. 4:**
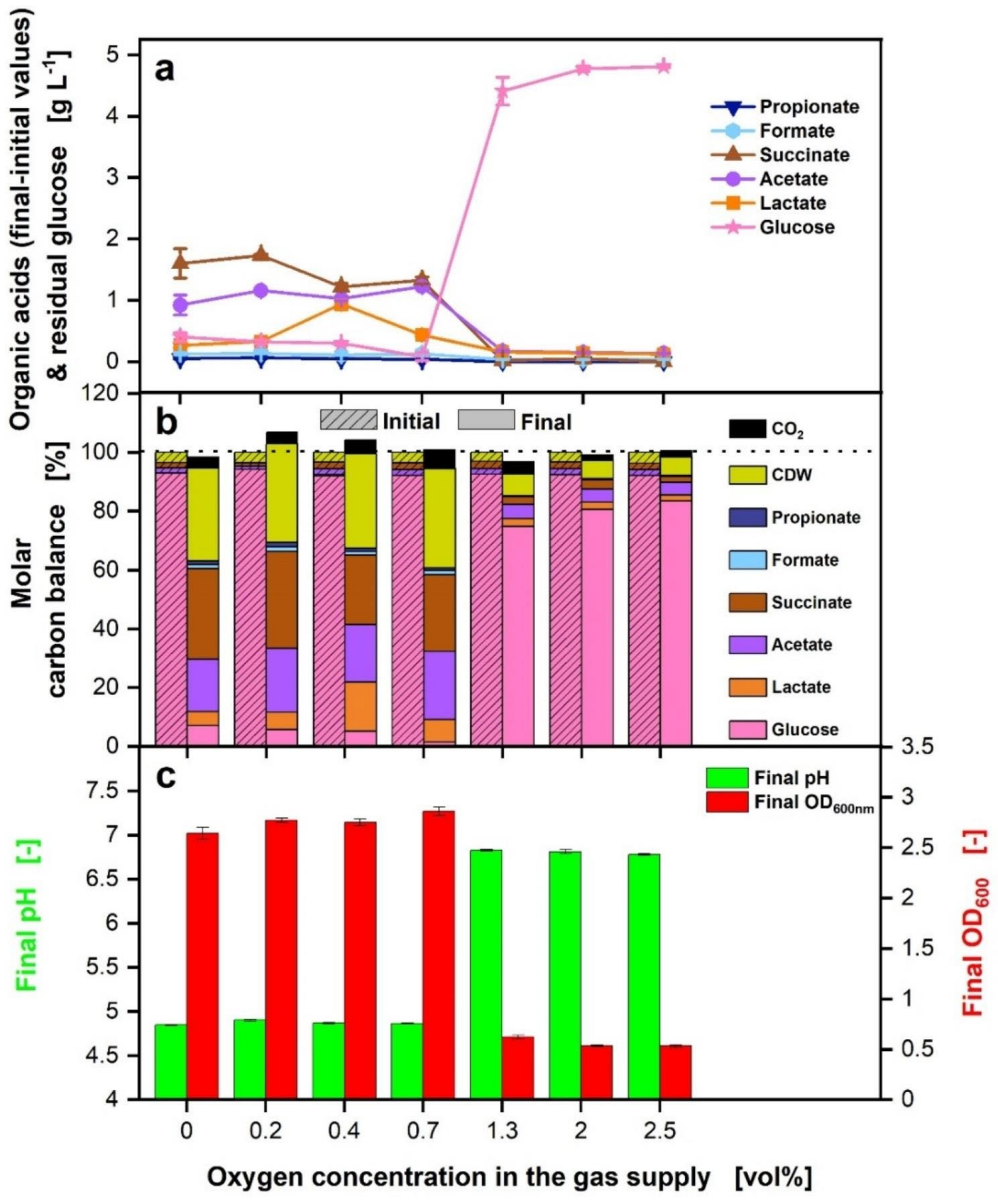
Effect of different O_2_ concentrations on offline data of *P. vulgatus* shake flask cultivations. These data refer to the experiment shown in Fig. 3. Offline data of (**a**) HPLC analysis of produced organic acids including propionate, formate, succinate, acetate and lactate and remaining glucose from four biological replicates with standard deviation. (**b**) Carbon balance in % as function of the O_2_ concentration in the gas supply. The start of the fermentation was set to 100%. Initial samples were drawn after inoculation. (**c**) Final OD_600nm_ and final pH from four biological replicates with standard deviation. Experimental setup is illustrated in Fig. 5b. Final OD_600nm_ and final pH each differed statistically significant for the different oxygen concentrations, for final OD_600nm_: *p* < 0.001 and for final pH: *p* < 0.001. Medium: DMM-G, c_Glucose_ = 6 g L^− 1^, c_buffer_ = 50 mM MOPS, T = 37 °C, n = 100 rpm, V_L_ = 50 mL, initial OD_600nm_ = 0.29, initial pH after inoculation = 6.9–7.1, vvm = 0.2 min^− 1^, different gas mixtures of O_2_ & 4% CO_2_ in N_2_, as indicated in the legend, N = 4

In Fig. 4, the offline data of this set of experiments are depicted. HPLC analysis of SCFAs and glucose is outlined in Fig. 4a. Almost no propionate (dark blue reverse triangles) and formate (light blue hexagons) are formed between 0 and 0.7 vol% O_2_, and production stops completely with higher O_2_ concentrations. Succinate concentrations (brown triangles) decrease slightly between 0.2 and 0.7 vol% O_2_. At more elevated O_2_ concentrations, the concentrations decline rapidly. Acetate concentrations (purple circles) remain constant between 0 and 0.7 vol% O_2_ and show a substantial decrease at higher O_2_ concentrations. Lactate production (orange squares) is highest at 0.4 vol% O_2_ and strongly lowers between 1.3 and 2.5 vol% O_2_. Almost no acids are formed at O_2_ concentrations higher than 0.7 vol%. The remaining glucose (pink stars) decreases between 0 and 0.7 vol% O_2_. Only low amounts of glucose have been consumed for O_2_ concentrations between 1.3 and 2.5 vol%. The molar carbon balance presented in Fig. 4b is closed with a maximal deviation of 6.5%. Cell dry weight (CDW) accounts on average for 32.8% of the total carbon for O_2_ concentrations between 0 and 0.7 vol% and 6.6% at O_2_ concentrations between 1.3 and 2.5 vol%. As in the set of experiments before, CO_2_ contribution to the total carbon is low, with a maximum of 6.4%. Figure 4c illustrates the final pH values and the final OD_600nm_. A Mann-Whitney-U-Test was conducted to assess the influence of different oxygen concentrations on the final OD_600nm_ and final pH values. Final OD_600nm_ and final pH values were split in two groups for the statistical analysis, low (0-0.7 vol%) and high oxygen concentration levels (1.3–2.5 vol%). The distributions between both groups differed, according to Kolmogorov-Smirnov *p* < 0.001. The final OD_600nm_ and final pH values differed statistically significant for the two oxygen concentration levels. The final pH values reveal a significant increase between 0.7 and 1.3 vol% O_2_, while the data of the final OD_600nm_ displays a substantial decrease between those O_2_ concentrations. To exclude the pH value as a responsible parameter for the observed results, the influence of different O_2_ concentrations was validated in another set of experiments with higher initial pH values (Fig. S3).

## Discussion

### Influence of CO_2_ on growth and organic acid production

With increasing CO_2_ concentration in the gas supply up to a concentration of 10.0 vol% (Fig. 1), CO_2_ production rises. However, interpretation of CO_2_ production via CTR must be conducted cautiously, as pH changes can also contribute to CTRs. While pH values decrease, CO_2_ is released due to the chemical CO_2_/HCO_3_^−^ balance [29]. During cultivation, the pH values decrease caused by the strain’s organic acid production. With increasing CO_2_ concentrations, especially observable from 3.0 vol%, the maximum of the CTR and TGTR curves is reached faster. This behavior can be observed until a CO_2_ concentration of 10.0 vol% is obtained. Lower CO_2_ concentrations, especially 0.25-1.0 vol%, prolong the lag phase of gas formation. Caspari and Macy [30] observed a prolonged lag phase for genetically related *Bacteroides fragilis* for CO_2_/HCO_3_^−^ concentrations below 10 mM (corresponding to about 12 vol% CO_2_ in the gas supply). They further observed that the maximum growth rate and cell yield decreased. Interestingly, the limiting CO_2_ concentrations observed for *B. fragilis* are substantially higher than those observed for *P. vulgatus* in this study. Furthermore, Franke and Deppenmeier [21] observed that *P. vulgatus* is less dependent on CO_2_ and HCO_3_^−^ as the genetically related *Prevotella copri*. While *P. copri* only reached maximal biomass formation above 20 mM HCO_3_^−^, *P. vulgatus* reached maximal growth yields at lower HCO_3_^−^ concentrations of ~ 10 mM. Reilly [25] studied the CO_2_ optimum for *P. vulgatus* cultivating on agar plates and found the optimum between 0.25 and 40 vol% CO_2_ in the gas supply. Above 40 vol% CO_2_, growth was inhibited. This study already observed reduced gas production at 15 vol% CO_2_.

The TGTR is higher than the CTR, confirming the formation of another gas besides CO_2_. Gas chromatography (data not shown) has proven that the only other gas besides CO_2_ is H_2_, which has also been revealed in other studies for *P. vulgatus* [31–33]. In Fig. S1, the hydrogen transfer rate (HTR) for 0.75 to 4.0 vol% CO_2_, calculated from TGTR and CTR, is plotted over the CTR. The decreasing slope with increasing CO_2_ concentration points out that while the CO_2_ concentration in the gas supply decreases, more H_2_ is formed, in relation to CO_2_. Since the metabolic pathways of *P. vulgatus* are not yet fully understood, it is unclear, what causes this shift from CO_2_ to H_2_ production.

Since at a concentration of 0.25 vol% CO_2_, the OD_600nm_ is lower than for the higher CO_2_ concentrations, some amount of CO_2_ seems to be utilized for biomass production. Caspari and Macy [30] could as well observe for *B. fragilis* a decreasing final OD_600nm_ for concentrations of ~ 12 vol% CO_2_ or lower in the gas supply. The pH (Fig. 2) decreases strongly at all CO_2_ concentrations higher than 0.25 vol%. Final values are in an inhibitory range for *P. vulgatus*. The literature demonstrates that pH values below 6.0 have a growth inhibitory effect on *P. vulgatus*, and growth stops entirely at a pH value below 5.3 [4, 34]. The second lowest final pH is reached at 3.0 vol% CO_2_, affiliating with the highest organic acid production. The lowest final pH is reached at 15.0 vol% CO_2_, probably caused by the high CO_2_ concentration, as it does not correlate with the highest organic acid production. The low final pH is a factor for growth inhibition, and another factor may be product inhibition by the organic acids formed by *P. vulgatus*.

At increasing CO_2_ concentrations, succinate, acetate, and formate production are rising (Fig. 2). Lactate formation increases with decreasing CO_2_ concentration. The high lactate formation at low CO_2_ concentrations may be an easy way for *P. vulgatus* to balance the production of redox equivalents [22]. Lactate production requires only the enzyme lactate dehydrogenase and is the most straightforward metabolic pathway [10]. Enhanced lactate production at lower CO_2_ concentrations and an increased acetate concentration at elevated CO_2_ concentrations were also observed in the study of Caspari and Macy [30] for *B. fragilis*. Succinate formation rises with increasing CO_2_ concentration in the gas supply, since the organism must first accumulate CO_2_ to start succinate formation [3]. Under CO_2_ deficiency, succinate could be converted to propionate, to release bound CO_2_ and utilize the CO_2_. However, in this study, no propionate could be detected with HPLC measurements, even at low CO_2_ concentrations, e.g., 0.25 vol%. At low CO_2_ concentrations, little succinate was produced, so almost no succinate was available for conversion to propionate. Acetate and formate do not increase as much as succinate with elevated CO_2_ concentration. Metabolic limitation is evident for all cultivations with CO_2_ concentrations below 3.0 or above 10.0 vol%, because glucose is not completely metabolized.

Although succinate production increased with increasing CO_2_ concentration, with 15.0 vol% CO_2_ in the gas supply, *P. vulgatus* achieved only 0.36 mol succinate/mol glucose. Therefore, *P. vulgatus* is less efficient than other succinate producers, such as *A. succinogenes* with 1.42 mol succinate/mol glucose [35] or *A. succiniciproducens* with 1.33 mol succinate/mol glucose [36]. *P. vulgatus* forms high amounts of acetate and lactate besides succinate and low amounts of formate, resulting in a yield for total SCFA of 1.1 mol acid/mol glucose. The total SCFA yield is close to the succinate yield of the beforementioned producers. The carbon balance is closed for these experiments, demonstrating that all major products contributing carbon to the balance have been considered. In this study, the optimal CO_2_ production for acid production by *P. vulgatus* was 3.0 vol%. Biomass growth was highest in the range of 0.5 to 4.0 vol%.

### Influence of O_2_ on growth and organic acid production

*P. vulgatus* reveals an unimpaired growth in the range of 0-0.7 vol% O_2_ in the gas supply (Fig. 3). Within this range, there is little deviation between all measured values, online and offline. An abrupt decline in viability is visible in the data, while increasing the O_2_ concentration from 0.7 to 1.3 vol%. This decline is evident for all measured values (Figs. 3 and 4). The exhibited O_2_ tolerance is higher than the published data of *Bacteroides* species, which specifies the O_2_ tolerance between 0.03 and 0.4 vol%. Only for *Bacteroides melaninogenicus* a higher O_2_ tolerance of up to 2.5 vol% was evaluated [15, 20]. It should be noted that the addition of L-cysteine as a reducing agent to the medium can reduce the oxidative damage on *P. vulgatus*. The relatively high O_2_ tolerance of *P. vulgatus* helps the species to maintain its high proportion in the gut, as O_2_ can be encountered in low concentrations of < 1 vol% [15, 37, 38]. However, the decline in viability, acid, and gas production above 0.7 vol% O_2_ is due to the damage that O_2_ causes to anaerobic bacteria. Molecular O_2_ diffuses into the cell and inactivates enzymes with a radical in its active center [15]. Another mechanism may be the formation of ROS, which can react with many molecules in the cell and lead to DNA, lipid, and disulfide bond damage [16].

As observed in the experiments with changing CO_2_ concentrations, the TGTR is substantially higher than the CTR. Comparing HTR with CTR (Fig. S2) discloses that the O_2_ concentration has only a neglectable influence on the CO_2_/H_2_ ratio, compared to the influence of the CO_2_ concentration in the gas supply.

Interestingly, despite the anaerobic character of *P. vulgatus*, a positive OTR (oxygen consumption) could be measured (Fig. 3). However, the OTR values are lower than those of aerobic microorganisms [27]. The OTR favor the assumption that O_2_ was reacting with medium components (e.g. L-cysteine) or was utilized in small amounts by *P. vulgatus* during the first hours of the cultivation. The enzyme cytochrome *bd* oxidase is a possible consumer of O_2_ [20], which enables the use of molecular O_2_ as a final electron acceptor instead of fumarate. In addition, cytochrome *bd* oxidase could act as a buffer enzyme to ensure electron flow through the anaerobic respiratory chain, despite O_2_ being present and prevent O_2_ from damaging other components in the cell. The study of Baughn and Malamy [20] finds evidence for unrestricted growth of *P. vulgatus* up to 0.03 vol% O_2_. The decrease in viability, when the O_2_ concentration reaches values above 0.7 vol%, is caused by the O_2_ damage exceeding the capacity of *P. vulgatus* protective mechanisms. In order to examine the oxidative stress response of *P. vulgatus*, L-cysteine should not be added to the medium. In this study, growth and organic acid production of *P. vulgatus* in a best-case scenario was investigated. Therefore, L-cysteine was added to the medium.

Considering the acid production (Fig. 4), succinate and propionate production decrease strongly with O_2_ concentrations of 0.7 vol% or higher. One possible explanation for this decrease is the O_2_-induced downregulation of the potentially ROS-forming enzyme fumarate reductase, which is necessary to produce succinate and propionate. For future experiments, expression studies for fumarate reductase are planned. The same behavior is observed for acetate production, as the enzymes crucial for acetate production are damaged by O_2_ [15]. The damaged acetate pathway leads to a lack of redox equivalents in the form of Fd_red_. The lack of redox equivalents could be the reason why lactate production is increased for 0.4 and 0.7 vol% O_2_. Lactate formation is an easy way for *P. vulgatus* to balance the production of redox equivalents [22].

The final pH is low, with a value of avg. 4.8 for the O_2_ range 0-0.7 vol% (Fig. 4c). The pH value influences *P. vulgatus* growth behavior. Therefore, the pH value as a responsible parameter for the observed results was excluded (Fig. S3). The slight increase in glucose consumption and final OD_600nm_ from 0 to 0.7 vol% O_2_ (Fig. 4a and c) lead to the conclusion that more glucose is used to produce biomass. Due to the impact of O_2_, the enzymes to produce SCFAs might be damaged or downregulated. Nevertheless, through the low amounts of produced SCFAs and the low growth, some metabolic activity is indicated even at O_2_ concentrations above 0.7 vol%. The activity probably occurred during the first hours of cultivation, when the protective mechanisms of *P. vulgatus* had not yet reached their capacity.

## Conclusions

Concluding this study, the optimum of tested CO_2_ concentrations for total organic acid and for succinate production by *P. vulgatus* is 3.0 vol% and 15.0 vol%, respectively. The O_2_ tolerance lies above 0.7, but below 1.3 vol%. *P. vulgatus* is inhibited by pH, the produced SCFAs, or a combination of both. The species could achieve higher titers of succinate in a pH-controlled fermentation, as demonstrated by Isar et al. [39] and Isar et al. [40] for *B. fragilis*. Other important next steps are genetic modifications, which have been proven to increase lactate production for *P. vulgatus* by Lück and Deppenmeier [10]. However, acid production must be shifted from acetate and lactate to succinate, the most valuable product. To evaluate the exact O_2_ tolerance of *P. vulgatus*, a continuous fermentation with a gradually raised O_2_ concentration over time would pose the best solution. Additionally, it would be important to have a medium without reducing agents, e.g. L-cysteine.

Determining the CO_2_ requirement and O_2_ tolerance for growth and organic acid production of *P. vulgatus* exhibits the potential for an industrial application. However, the species cannot yet compete with established industrial SCFA producers. The species requires little CO_2_ and has a certain O_2_ tolerance. These results may contribute to a faster optimization of *P. vulgatus* as an organic acid producer and display that strictly anaerobic bacteria can tolerate more O_2_ than expected.

## Methods

### Strain and media

The research group of Prof. Deppenmeier (Rheinische Friedrich-Wilhelms-Universität, Bonn, Germany) kindly provided the strain *Phocaeicola vulgatus* DSM 1447, obtained from the German Collection of Microorganisms and Cell Cultures (DSMZ, Braunschweig, Germany). Brain heart infusion medium (BHI) for cryogenic stocks was acquired as BD Difco™ (Thermo Fisher, Waltham, USA). BHI powder contained: 7.7 g L^− 1^ calf brain extract, 9.8 g L^− 1^ beef heart extract, 10 g L^− 1^ protease peptone, 2 g L^− 1^ dextrose, 5 g L^− 1^ sodium chloride, and 2.5 g L^− 1^ disodium phosphate, dissolved in deionized water. An active growing BHI culture was used to prepare cryogenic stocks after 24 h of cultivation by mixing 50 vol% culture broth with 50 vol% anaerobic sucrose solution (500 g L^− 1^) and freezing 1.8 mL aliquots at -80 °C. For all main and precultures, a defined minimal medium with glucose (DMM-G) was used. DMM-G composition was based on Varel and Bryant [41] and Lück and Deppenmeier [10] with 3-(*N*-morpholino)propanesulfonic acid (MOPS) buffer instead of bicarbonate buffer. If not stated otherwise, DMM-G medium components were obtained from Carl Roth (Karlsruhe, Germany). The medium consisted of 13 individual stock solutions: Base components (pH 7.4), glucose, calcium chloride, magnesium chloride, iron(II) sulfate, SL6-trace elements, Wolin’s vitamin solution, butyrate, vitamin K1, hemin, resazurin (Thermo Fisher, Waltham, USA), L-cysteine hydrochloride, and MOPS buffer (pH 7.4). Stock solutions were stored separately, as premature mixing would have caused precipitation. The base components stock comprised ammonium chloride, dipotassium phosphate, monopotassium phosphate, and sodium chloride. The SL6-trace elements included boric acid, cobalt(II)chloride hexahydrate, copper(II)chloride dihydrate, manganese(II)chloride tetrahydrate (Merck, Darmstadt, Germany), nickel(II)chloride, sodium molybdate dihydrate and zinc sulfate heptahydrate (Merck, Darmstadt, Germany) and were set to pH 7.4 with 5 M sodium hydroxide. The Wolin’s vitamin stock solution contained α-lipoic acid, biotin, folate (Sigma Aldrich, St. Louis, USA), nicotinamide, p-aminobenzoic acid (Sigma Aldrich, St. Louis, USA), pantothenic acid (AppliChem, Darmstadt, Germany), pyridoxine hydrochloride (Sigma Aldrich, St. Louis, USA), riboflavin (Sigma Aldrich, St. Louis, USA), thiamine hydrochloride and vitamin B12. Table S1 lists the final concentrations of all components in the DMM-G medium. Base components, glucose, calcium chloride, magnesium chloride, iron(II) sulfate, and SL6-trace elements stocks were sterilized at 121 °C for 20 min. The remaining heat-sensitive stock solutions were sterile-filtered with 0.22 μm polyethersulfone filters (Merck, Darmstadt, Germany). To prevent premature oxidation, reducing agent L-cysteine was sterile-filtered and stored anaerobically in a serum bottle with a nitrogen atmosphere. Wolin’s vitamin solution, vitamin K1, hemin, and resazurin stock solutions were stored light-protected at 4 °C after sterilization. All other stock solutions were stored at room temperature.

### Cultivation conditions

Precultures were grown in serum bottles with a total volume of 250 mL. The serum bottles were filled with 50 mL DMM-G medium and sealed gas-tight with a rubber stopper and clamp. Afterward, the serum bottles were gassed with N_2_ for 20 min to ensure an anaerobic atmosphere. In the next step, CO_2_ was added to the serum bottles with a sterile syringe to obtain a CO_2_ headspace concentration of 10 vol%. Afterward, 0.1 mL L-cysteine solution was added as a reducing agent, and in the final step, the medium was inoculated with 500 μL cryogenic culture, both with a sterile syringe. The serum bottles were inoculated in a temperature-controlled shaker for 24 h at 37 °C with a shaking diameter of 50 mm and a shaking frequency of 100 rpm. The main experiments were performed in a RAMOS device designed by Anderlei and Büchs [27]. The RAMOS is a non-invasive online monitoring device for measuring CO_2_, O_2,_ and pressure for up to eight shake flasks. Anderlei and Büchs [27], Anderlei et al. [28], and Munch et al. [26] provide a schematic overview of the RAMOS setup and gas measurement phases as well as the calculation of the carbon dioxide transfer rate (CTR), oxygen transfer rate (OTR) and total gas transfer rate (TGTR). Measurement of the increase of produced gases is conducted with pressure sensors (26PCA, Honeywell, Charlotte, USA) and infrared carbon dioxide sensors (MSH-*P* − CO2, 126 Dynament, Mansfield, UK). The RAMOS device is a proven system and has already been operated with syngas [42] or ethylene [43] in the ingas. As the gas measurement phases needed to be adapted to the specific microorganism, time and gas flows were set for both CO_2_ and O_2_ experiments as follows: 20 min measurement phase without gas flow, 2.38 min high gas flow rate at 22.5 mL min^− 1,^ and 40 min low gas flow rate at 10 mL min^− 1^. Before inserting the shake flasks in the RAMOS device, they were filled with 45 mL sterile DMM-G medium and gassed overnight with the respective cultivation gas at 37 °C in a shaker (ISF1-X, Adolf Kühner AG, Birsfelden, Switzerland) at 100 rpm, with a shaking diameter of 50 mm. The system was tested for gas tightness to ensure anaerobic conditions and to prevent false gas measurements. As a reducing agent, 0.1 mL L-cysteine was inserted with a sterile syringe into each flask before inoculation with 5 mL preculture. Initial samples were drawn after inoculation, and final samples at the end of the cultivation.

### Gas mixing system

The gas mixing system consists of up to four mass flow controllers (MFCs) and one control unit, which can be connected to the RAMOS. Therefore, the signal from the RAMOS controls the gas mixing system, to switch between the aforementioned different gas measurement phases.

**Fig. 5:**
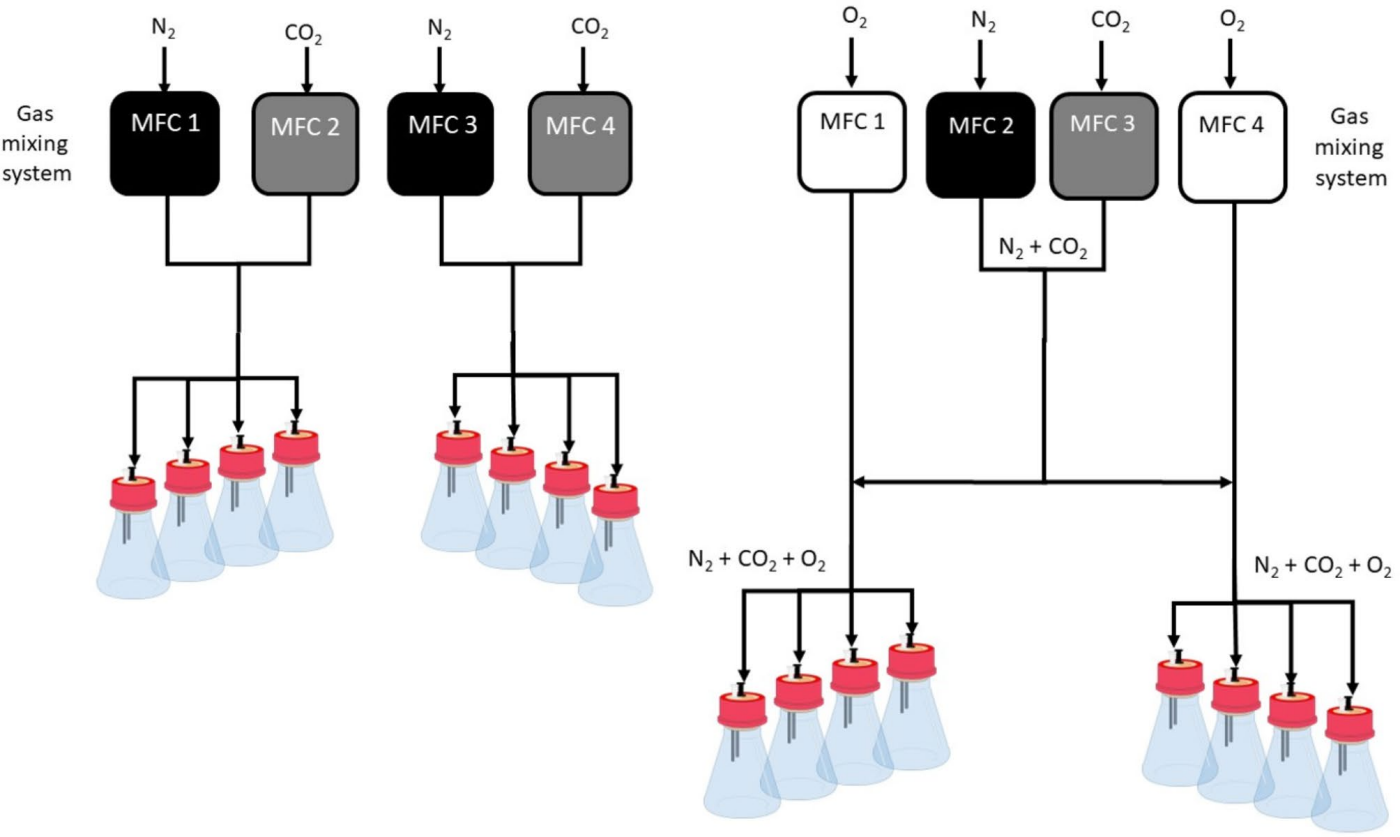
Schematic illustration of the experimental setup of the gas mixing system. Change of the (**a**) CO_2_ or (**b**) O_2_ concentration in the gas supply. In case of (**b**), the dilution of N_2_ and CO_2_ by O_2_ remains very low. Four mass flow controllers (MFC) were used with following ranges, for (**a**): MFC 1 & 3: 50–500 mL/min (calibrated with N_2_), MFC 2: 5–50 mL/min (calibrated with O_2_), MFC 4: 0.5-5 mL/min (calibrated with N_2_) and for (**b**): MFC 1: 5–50 mL/min (calibrated with O_2_), MFC 2 & 3: 50–500 mL/min (calibrated with N_2_) and MFC 4: 2–20 mL/min (calibrated with N_2_). This setup was chosen, as experiments at two different gas compositions can be performed with four shake flasks each

The schematic setup of the gas mixing system with gas supply lines can be found in Fig. 5a for different CO_2_ concentrations and Fig. 5b for different O_2_ concentrations. The setup was designed so that four shake flasks within the RAMOS can be operated with one gas concentration and the other four with a second gas concentration. After adjusting the gas supply lines, the gas flows were set prior to the experiments. Desired gas concentrations were configured as a percentage of the total maximum flow of the MFC on the control unit. Afterward, the settings of the MFCs were tested by measuring the total flow from the gas mixing system with a gas flow calibrator, Defender 530 + L (Mesa Laboratories, Inc., Lakewood, USA). Before the experiment, a calibration curve was created for the CO_2_ and O_2_ sensors within the RAMOS device. With the help of the calibration curve, the concentrations set by the gas mixing system of CO_2_ and O_2_ were checked and, if necessary, adjusted.

### Hydrogen transfer rate

Besides CO_2_, also H_2_ is produced. As no other gases are formed, the hydrogen transfer rate (HTR) was calculated by subtracting the CTR from the TGTR.

### Offline analysis

Initial and final samples were collected and directly used for OD_600nm_ measurement at a wavelength of 600 nm with a Genesys 20 spectrophotometer (Thermo Scientific, Germany). Samples were diluted with 9 g L^− 1^ NaCl. To correlate the optical density and CDW, the equation $CDW = 0.563 \cdot O{D_{600nm}}$, derived in [44, in revision] for *P. vulgatus*, was used. Samples not used for optical density measurement were centrifuged at 18,000 rpm for 5 min. The supernatant was used for HPLC and pH measurement. The pH was measured with a pH electrode (Mettler-Toledo, Columbus, USA). The remaining sample supernatant was stored at -80 °C for further HPLC analysis. Therefore, samples were thawed and filtered with 0.2 μm cellulose acetate filters (Merck, Darmstadt, Germany). The SCFAs, acetate, succinate, lactate, propionate, formate, and remaining glucose were measured by HPLC. The HPLC device (Dionex, Sunnyvale, USA) was equipped with an organic acid resin column of 300 × 8 mm dimensions (CS-Chromatography, Langerwehe, Germany) and set to 60 °C. As an eluent, 5 mM H_2_SO_4_ at a flow rate of 0.8 mL min^−1^ was applied. UV/VIS and a refractive index detector were used during HPLC measurement.

### Carbon balances

Carbon balances were calculated for all experiments with the following Eq. 1:

1 $$Carbo{n_{inX}}\left[ {\frac{{mmol}}{L}} \right] = \frac{{Carbon\,molecule{s_{inX}}\left[ - \right]}}{{{M_X}\left[ {\frac{g}{{mmol}}} \right]}} \cdot {c_X}\left[ {\frac{g}{L}} \right]$$

Where *X* is the specific compound, *c* is the concentration [g L^− 1^], *M*_*X*_ is the molar mass of the specific compound [g mol^− 1^], *Carbon molecules*_*in X*_ is the number of carbon atoms in the specific compound [-], and *Carbon*_*in X*_ is the molar carbon concentration for the compound [mmol L^− 1^].

The compounds glucose, acetate, lactate, succinate, propionate, formate, CO_2,_ and biomass of every sample were considered. Initial and final concentrations of glucose, acetate, lactate, succinate, propionate, and formate were measured by HPLC. The microbial biomass of *P. vulgatus* cells was based on data from Franke and Deppenmeier [21] of *P. copri* microbial biomass. Molar carbon from CO_2_ was calculated from the CTR integral based on equations in Munch et al. [26]. First, the volumetric molar carbon [mmol L^− 1^] for each compound was calculated, and then the values were combined to obtain the total volumetric molar carbon content for every sample. Finally, to achieve relative values for the carbon content of the compounds, the molar carbon value was divided by the total carbon of the sample, as shown in Eq. 2:

2 $$Carbo{n_{Samplen}}\left[ \% \right] = \frac{{Carbo{n_{in\,X,Sample\,n}}\left[ {\frac{{mmol}}{L}} \right]}}{{Total\,Carbo{n_{Sample\,n}}\left[ {\frac{{mmol}}{L}} \right]}}$$

Where *Sample n* is designated to a specific sample number in a specific experiment, *Carbon*_*in X, Sample n*_ is the volumetric molar carbon of the specific compound in *Sample n* [mmol L^− 1^], and *Total Carbon*_*Sample n*_ is the sum of all carbon in this *Sample n* [mmol L^− 1^].

### Software

All graphs were created with OriginPro® version 2021 from OriginLab Corporation (Massachusetts, USA).

### Statistical analyses

Statistical Analyses were performed in order to assess the influence of different oxygen concentrations on different cultivation parameters by Mann-Whitney-U-Test using OriginPro® version 2021 from OriginLab Corporation (Massachusetts, USA). Final OD_600nm_ and final pH values were split in two groups for the statistical analysis, low (0-0.7 vol%) and high oxygen concentration levels (1.3–2.5 vol%). To determine if the distributions between both groups differed, the Kolmogorov-Smirnov-Test was conducted.

### Electronic supplementary material

Below is the link to the electronic supplementary material.

**Supplementary Material 1: Fig. S1** HTR plotted over CTR with linear fit for *P. vulgatus* cultivations with changing CO2 in the gas supply

**Supplementary Material 2: Fig. S2** HTR plotted over CTR with linear fit for *P. vulgatus* cultivations with changing O2 in the gas supply

**Supplementary Material 3: Fig. S3** Effect of changing initial pH value and changing oxygen concentrations in the gas supply

**Supplementary Material 4: Table S1**: Concentration of DMM-G medium components used in this work in alphabetical order

## Data Availability

The datasets generated during and/or analyzed during the current study are available from the corresponding author upon reasonable request.
