# Supplementary material for: Carbon dioxide and trace oxygen concentrations impact growth and product formation of the gut bacterium Phocaeicola vulgatus
Source: BMC Microbiol. 2023 Dec 7;23:391. doi: 10.1186/s12866-023-03127-x (PMC10701953; doi:10.1186/s12866-023-03127-x)
Supplement: Supplementary file 1 — Supplementary Material 1: Fig. S1 HTR plotted over CTR with linear fit for P. vulgatus cultivations with changing CO2 in the gas supply [file 12866_2023_3127_MOESM1_ESM.docx]

**Fig. S1 HTR plotted over CTR with linear fit for *P. vulgatus* cultivations with changing CO_2_ in the gas supply.** Hydrogen transfer rate (HTR) is shown over CTR for (a) 4%, (b) 3%, (c) 1% and (d) 0.75% CO_2_ in the gas supply. HTR was calculated from TGTR-CTR with standard deviation from four biological replicates. Data was obtained from the experiment shown in Fig. 2. For CO_2_ concentrations in the gas supply of 0.5% and 0.25% no reasonable plot was obtained. For 10% and 15%, no CTR could be measured. Medium: DMM-G, c_Glucose_ = 6 g L^-1^, c_buffer_ = 50 mM MOPS, T = 37 °C, n = 100 rpm, V_L_ = 50 mL, initial OD_600nm_ = 0.2, initial pH after inoculation = 6.9-7.15, vvm = 0.2 min^-1^, different gas mixtures = CO_2_ in N_2_
