# Supplementary material for: Carbon dioxide and trace oxygen concentrations impact growth and product formation of the gut bacterium Phocaeicola vulgatus
Source: BMC Microbiol. 2023 Dec 7;23:391. doi: 10.1186/s12866-023-03127-x (PMC10701953; doi:10.1186/s12866-023-03127-x)
Supplement: Supplementary file 2 — Supplementary Material 2: Fig. S2 HTR plotted over CTR with linear fit for P. vulgatus cultivations with changing O2 in the gas supply [file 12866_2023_3127_MOESM2_ESM.docx]

**Fig. S2 HTR plotted over CTR with linear fit for *P. vulgatus* cultivations with changing O_2_ in the gas supply.** Hydrogen transfer rate (HTR) is shown over CTR for (**a**) 0%, (**b**) 0.2%, (**c**) 0.4%, (**d**) and (**e**) 0.7% O_2_ in the gas supply. HTR was calculated from TGTR-CTR with standard deviation from four biological replicates. The OTR was neglected, due to its very low values in this range of oxygen concentrations. Data was obtained from the experiment shown in Fig. 4 and Fig. S3. Symbol * indicates elevated initial pH after inoculation of 7.18, deviating from the other initial pH values in this experiment. For larger O_2_ concentrations in the gas supply than 0.7%, no reasonable parity plots were obtained. Medium: DMM-G, c_Glucose_ = 6 g L^-1^, c_buffer_ = 50 mM MOPS, T = 37 °C, n = 100 rpm, V_L_ = 50 mL, initial OD_600nm_ = 0.2, initial pH after inoculation = 6.96-7.15, vvm = 0.2 min^-1^, different gas mixtures = O_2_ and 4% CO_2_ in N_2_, N = 4
