# Supplementary material for: Carbon dioxide and trace oxygen concentrations impact growth and product formation of the gut bacterium Phocaeicola vulgatus
Source: BMC Microbiol. 2023 Dec 7;23:391. doi: 10.1186/s12866-023-03127-x (PMC10701953; doi:10.1186/s12866-023-03127-x)
Supplement: Supplementary file 3 — Supplementary Material 3: Fig. S3 Effect of changing initial pH value and changing oxygen concentrations in the gas supply [file 12866_2023_3127_MOESM3_ESM.docx]

**Fig. S3 Effect of changing initial pH value and changing oxygen concentrations in the gas supply.** Online data of (**a**) carbon dioxide transfer rate (CTR) and (**b**) total gas transfer rate (TGTR), (**c**) oxygen transfer rate (OTR). Shadows indicate standard deviations of four biological replicates. Offline data of (**d**) produced organic acids including propionate, formate, succinate, acetate, lactate and remaining glucose, (**e**) final OD_600nm_ and final pH. In (**d**) and (**e**) standard deviation of four biological replicates is shown. Experimental setup is illustrated in Fig. 1**b**. Results shown here correspond to results shown in Figure 4. Medium: DMM-G, c_Glucose_ = 6 g L^-1^, c_buffer_ = 50 mM MOPS, T = 37 °C, n = 100 rpm, V_L_ = 50 mL, initial OD_600nm_ = 0.29, initial pH after inoculation = 6.96-7.18, vvm = 0.2 min^-1^, different gas mixtures of O_2_ at 4 % CO_2_ in N_2_
