# Supplementary material for: Carbon dioxide and trace oxygen concentrations impact growth and product formation of the gut bacterium Phocaeicola vulgatus
Source: BMC Microbiol. 2023 Dec 7;23:391. doi: 10.1186/s12866-023-03127-x (PMC10701953; doi:10.1186/s12866-023-03127-x)
Supplement: Supplementary file 4 — Supplementary Material 4: Table S1: Concentration of DMM-G medium components used in this work in alphabetical order [file 12866_2023_3127_MOESM4_ESM.docx]

| Name | Final concentration in DMM-G medium |
| --- | --- |
| α-lipoic acid | 0.05 mg L^-1^ |
| Ammonium chloride | 0.75 g L^-1^ |
| Biotin | 0.02 mg L^-1^ |
| Boric acid | 0.6 mg L^-1^ |
| Butyric acid | 0.176 mg L^-1^ |
| Calcium chloride | 0.026 g L^-1^ |
| Cobalt(II)chloride hexahydrate | 0.4 mg L^‑1^ |
| Copper(II)chloride dihydrate | 0.02 mg L^-1^ |
| Dipotassium phosphate | 2.2 g L^-1^ |
| Folate | 0.02 mg L^-1^ |
| Glucose | 6.0 g L^-1^ |
| Hemin | 1.0 mg L^-1^ |
| Iron(II)sulphate | 1.39 mg L^-1^ |
| L-cysteine hydrochloride | 484 mg L^-1^ |
| Magnesium chloride | 0.1 g L^-1^ |
| Manganese(II)chloride tetrahydrate | 0.06 mg L^-1^ |
| Monopotassium phosphate | 1.7 g L^-1^ |
| Nickel(II)chloride hexahydrate | 0.04 mg L^-1^ |
| Nicotinamide | 0.05 mg L^-1^ |
| P-aminobenzoic acid | 0.05 mg L^-1^ |
| Pantothenic acid | 0.05 mg L^-1^ |
| Pyridoxine hydrochloride | 0.1 mg L^-1^ |
| Riboflavin | 0.05 mg L^-1^ |
| Sodium chloride | 0.9 g L^-1^ |
| Sodium molybdate dihydrate | 0.06 mg L^-1^ |
| Thiamine hydrochloride | 0.05 mg L^-1^ |
| Vitamin B12 | 0.001 mg L^-1^ |
| Vitamin K1 | 9.95 ·10^−5^ vol% |
| Zinc sulphate heptahydrate | 0.2 mg L^-1^ |

**Table S1: Concentration of DMM-G medium components used in this work in alphabetical order.**
